# Standard formulas and individualised parenteral nutrition preparations in very low birth weight infants

**DOI:** 10.3389/jpps.2025.15310

**Published:** 2025-11-19

**Authors:** Laurie Dez, Stéphane Haÿs, Gilles Leboucher, Romain Garreau, Jean-Charles Picaud, Thomas Briot

**Affiliations:** 1 Pharmacy Department, Hospices Civils de Lyon, Groupement Hospitalier Nord, Lyon, France; 2 Neonatology Department, Hospices Civils de Lyon, Groupement Hospitalier Nord, Lyon, France; 3 Laboratoire de Biométrie et Biologie Évolutive, UMR CNRS 5558, Université Claude Bernard Lyon 1, Villeurbanne, France; 4 CarMen, INSERM U1060, INRA U197, Université Claude Bernard Lyon 1, Villeurbanne, France; 5 LAGEPP, CNRS UMR5007, Université Claude Bernard Lyon 1, Villeurbanne, France

**Keywords:** neonatology, parenteral nutrition, standardisation, pharmacy, recommendations

## Abstract

**Background/objectives:**

Optimal nutrition in very low birth weight (VLBW) infants is associated with improved clinical outcomes. When parenteral nutrition (PN) with a marketing authorisation is not appropriate, hospital pharmacies can prepare more suitable PN preparation. This corresponds to standard preparations (i.e., available at any time with a fixed composition) or individualised ones (i.e., available after a period of prescription, preparation, and pharmaceutical control). In France, 12 standard formulas to be compounded were proposed by a national consortium in 2018. The objective of the present study was to evaluate whether individualised PN preparations ordered in our hospital are substitutable by one of the 12 standard formulas.

**Methods:**

All PN prescriptions for VLBW infants made in 2021 in our hospital were retrospectively extracted. For each prescription, the theoretical intakes that an infant would have received if a standard preparation had been administered were calculated. Standard and individualised preparations were compared using the Mann-Whitney U test for each component. Secondly, the relative difference between the expected intakes and effectively intakes was calculated for each component.

**Results/Discussion:**

Over the study period, 1708 prescriptions were identified (corresponding to 1708 PN individualised preparations). Most infants were extremely low birth weight infants. Based on the methods of comparison, none of the 12 standard formulas fitted with targeted intakes achieved with individualised PN preparations ordered, whereas prescriptions did fit with international guidelines.

**Conclusion:**

The study highlights how it is difficult to establish nationally standard PN formulas for VLBW infants; the development of local standard formulas seems therefore relevant.

## Introduction

Extremely preterm infants are at high risk of neurodevelopmental delay [1, 2] and an optimal nutrition has been associated with improved neurodevelopmental outcomes and morbidity-free survival [3, 4]. Nutritional intake must follow guidelines and should start as early as possible after birth with the objective to achieve post-natal growth rate similar to that of the foetus [5].

Concerning parenteral nutrition (PN) preparations, they are classically grouped into three categories: preparations with a marketing authorisation, standard preparations or individualised preparations. While PN preparations with a marketing authorisation are produced on a large scale by pharmaceutical industries, standard and individualised preparations are produced by hospital pharmacies or by authorized pharmaceutical establishments. Standard preparations are made in small batches for several patients and compounded at least weeks before administration to patients. Individualised preparations are generally adapted daily to fit with specific patient needs and are extemporaneously compounded.

In 2018, the European Society for Paediatric Gastroenterology Hepatology, and Nutrition (ESPHGAN) recommended the use of standard rather than individualised PN preparations for the majority of paediatric and neonatal patients, including those with very low birth weight (VLBW) [6]. In the specific case of VLBW neonates, who are particularly difficult to manage, studies have demonstrated that optimal nutrition improves weight gain and minimises the length deficit at discharge whether the PN is individualised preparations or standard preparations with supplementation of amino acids [7, 8]. The goal of standard PN is to improve patients’ safety by minimising procedural incidents and optimising resource efficiency while providing clinically appropriate nutrition that meets individual patient’s needs [9]. In specific cases, it has also been demonstrated that standard PN improved daily intake, notably in amino acid intakes, as compared to individualised PN [10, 11]. According to the 2018 French health authority (*Haute Autorité de Santé*, HAS) recommendations, the type of PN for newborns should be chosen based on the patient’s nutritional needs and depends on the availability of a hospital pharmacy to produce PN [12]. The use of preparations with marketing authorization is recommended in first intention, given their maximum level of safety regarding microbiology risk notably. When the needs of a patient cannot be covered by the PN preparation with a marketing authorisation, standard PN preparations are then recommended. When neither a preparation with a marketing authorisation nor a standard preparation is suitable, an individualised preparation produced in a hospital pharmacy may be prescribed [12, 13]. In 2018, at the request of the French Directorate of Health Care Supply (*Direction Générale de l’Offre de Soins*, DGOS) and the Directorate General of Health, Ministry of Solidarities and Health (*Direction Générale de la Santé*, DGS), a national consortium was constituted to establish a limited number of standard formulas to be compounded that can be used in a wide range of neonates [12]. The working group included six neonatalogists and seven hospital pharmacists. The composition of the proposed standard PN formulas had to align with international guidelines, feedback from international standardisation efforts, insights from French hospitals using standard preparations, and an analysis of 19,000 individualised preparations previously compounded in France. This group established 12 formulas to be included in the National Formulary of the French Pharmacopoeia [12].

The objective of the present study was to evaluate whether individualised PN preparations ordered for VLBW infants in our hospital are substitutable by one of the 12 standard PN formulas proposed by the national consortium.

## Materials and methods

### Study population

Individualised PN preparations compounded by the pharmacy department at the Croix-Rousse University Hospital for VLBW admitted to the level 3 neonatal intensive care unit (NICU) during 2021 were first identified. Then, all corresponding PN prescriptions (without any exclusion criterion) were retrospectively extracted from electronic medical records using IntelliSpace Critical Care & Anesthesia [v J.00.010] software (Philips Medical Systems, Andover, MA, United States). All prescriptions are electronic and automatically included in the electronic medical records. Gestational age, age (day of life) at prescription, patient’s weight, the volume prescribed (mL/kg/day), and all intakes prescribed were collected. Intakes correspond to amino acid (g/kg/day), carbohydrates (g/kg/day), lipids (g/kg/day), sodium (mmol/kg/day), potassium (mmol/kg/day), calcium (mmol/kg/day), magnesium (mmol/kg/day), phosphorus (mmol/kg/day), trace elements (mL/kg/day; Junimin®, Aguettant, Lyon, France) and vitamins (mL/day; Cernevit®, Baxter, Guyancourt, France).

This single-centre, descriptive, retrospective study was approved by the local Scientific and Ethics Committee of the Hospices Civils de Lyon (*Comité Scientifique et Éthique des Hospices Civils de Lyon*, number 22-5054).

As nutritional intakes have to be adapted to the day of life of VLBW, individualised prescriptions (i.e., corresponding to compounded PN) were then separated into three subgroups according to their day of life (D). Groups were defined according to international guidelines that recommend initiating parenteral nutrition as early as possible on the first day of life, followed by a gradual increase in fluid and macronutrient intakes until a plateau is typically reached after 3–5 days [5]. Group D_0_ corresponded to prescriptions at D0 (i.e., day of birth), Group D_1–3_ to prescriptions from D1 to D3, and Group D_4+_ to prescriptions from D4 onward.

### Calculation of the expected intakes with the standard preparation

For each individualised PN prescription, the intakes that an infant would have received if a standard preparation had been administered were calculated. This was done for each of the 12 standard formulas (Asphystart®, Metabstart®, Premconc®, Premconc L®, Premend®, Premend L®, Premgo®, Premgo L®, Premstard 20®, Premstart 30®, Termgo®, and Termstart®; HAS). The volume of the standard preparation was identical to that of the individualised preparation. If an individualised prescription contained lipids and the standard preparation did not, the volume taken into consideration was that of the individualised prescription minus that specifically provided by lipids assuming the use of a 20% lipid emulsion.

### Comparison of individualised preparation with standard preparations

Demographic variables at inclusion were described by mean ± standard deviation (sd), or median interquartile range [IQR] as appropriate. Intakes were compared between the individualised PN prescriptions and the theoretical intakes of each standard preparation.

Initially, all components of PN preparation were compared using the Mann-Whitney U test. The p-value was adjusted using Bonferroni correction, and an adjusted p-value <0.05 was considered significant. Analyses were performed using R statistical software (version 4.3.1; R Core Team; R Foundation for Statistical Computing, Vienna, Austria). Individualised preparations were considered to be substitutable by standard one in the absence of significant difference for all components.

The relative difference between the expected intakes of each standard preparation and the individualised PN preparation was calculated for each component. An individualised preparation was considered substitutable by a standard preparation if the difference was <20% for each component. In the absence of established guidelines regarding an acceptable difference, this threshold was empirically determined by extrapolating from the definition of bioequivalence where the 90% confidence interval for the two compared products lie within 80–125% acceptance range for AUC_0–t_ and Cmax.

## Results

### Study population

**FIGURE 1:**
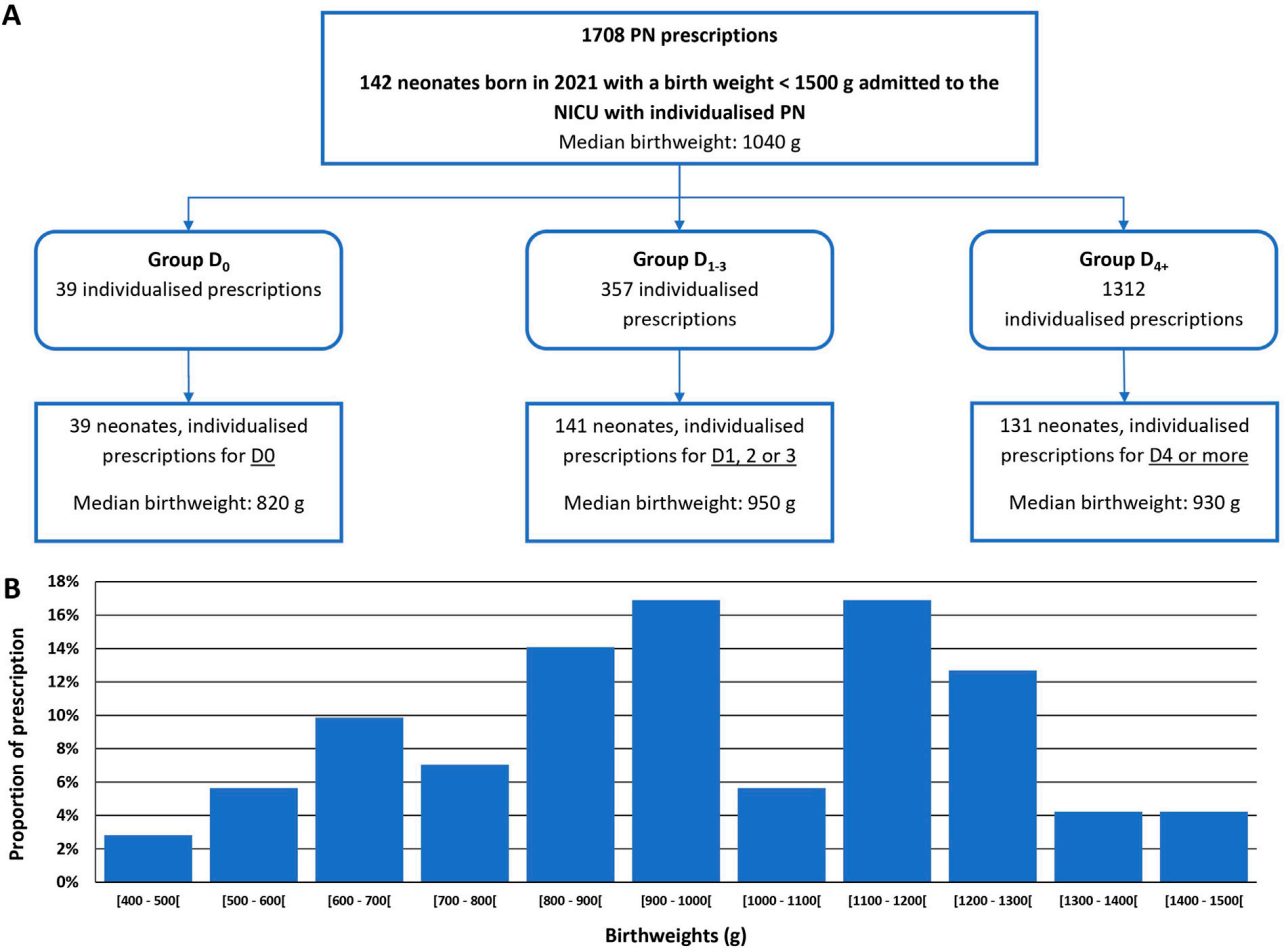
Study flow chart **(A)** and graphical representation of birthweight dispersion of the 142 neonates admitted **(B)**. NICU: Neonatal intensive care unit PN: parenteral nutrition.

Over the study period, 1708 individualised PN prescriptions were included (corresponding to 142 individual neonates; Figure 1A). Among the 142 neonates, 56% were extremely low birth weights infants (ELBW; Figure 1B).

The majority (76.8%) of PN prescriptions were for D4 onward (Group D_4+_). Prescriptions for preterm infants aged from 1 to 3 days (Group D_1-3_) represented 20.9% of the total prescriptions, and 2.3% corresponded to prescriptions for the day of birth (Group D_0_). Gestational ages were comprised between 24weeks +1 day and 32weeks +1 day (Table 1). The median [IQR] compositions of individualised PN compounded for each group are detailed in Table 2.

**TABLE 1:** Characteristics of the 142 neonates at birth.

Parameter	Group D_0_	Group D_1-3_	Group D_4+_
Gestational age, weeks + days	27 + 4 [26 + 2 – 29 + 4]	28 + 4 [26 + 3 – 29 + 5]	28 + 2 [26 + 2 – 29 + 5]
Gestational age, weeks + days (range)	(24 + 1 – 32 + 1)	(23 + 6 – 32 + 1)	(23 + 6 – 32 + 1)
Birthweight, grams	820 [630 – 1005]	950 [780 – 1160]	930 [775 – 1140]
Birthweight, grams (range)	(480 – 1260)	(441 – 1490)	(441 – 1470)

Data are given as median [IQR] unless otherwise stated.

**TABLE 2:** Intakes as ordered to the pharmacy for each group.

Nutrient	Group D_0_	Group D_1-3_	Group D_4+_
IV fluid intake, mL/kg/day	64.0 [60.0 – 72.0]	79.0 [66.0 – 93.0]	85.0 [69.0 – 100.3]
Amino acids, g/kg/day	2.0 [2.0 – 2.0]	2.5 [2.2 – 3.0]	3.0 [2.5 – 3.5]
Carbohydrates, g/kg/day	8.0 [7.0 – 8.0]	11.0 [9.0 – 12.0]	14.0 [12.0 – 16.0]
Lipids, g/kg/day	0.5 [0.5 – 0.5]	1.5 [1.0 – 2.0]	2.0 [2.0 – 2.5]
Sodium, mmol/kg/day	0.3 [0.3 – 0.5]	2.0 [1.0 – 3.0]	5.0 [4.0 – 6.0]
Potassium, mmol/kg/day	0.5 [0.2 – 0.5]	1.0 [0.8 – 1.5]	1.5 [1.0 – 2.0]
Calcium, mmol/kg/day	1.0 [1.0 – 1.0]	1.0 [1.0 – 1.2]	1.0 [0.8 – 1.0]
Magnesium, mmol/kg/day	0.3 [0.3 – 0.3]	0.3 [0.3 – 0.3]	0.3 [0.3 – 0.3]
Phosphorus, mmol/kg/day	0.3 [0.2 – 0.5]	1.2 [0.8 – 1.5]	2.0 [1.5 – 2.0]
Trace elements, mL/kg/day	1.0 [1.0 – 1.0]	1.0 [1.0 – 1.0]	1.0 [1.0 – 1.0]
Vitamins, mL/day	0.0 [0.0 – 0.0]	1.0 [1.0 – 1.0]	1.0 [1.0 – 1.0]

Data are given as median [IQR].

### Calculation of the expected intakes with a standard preparation

**FIGURE 2:**
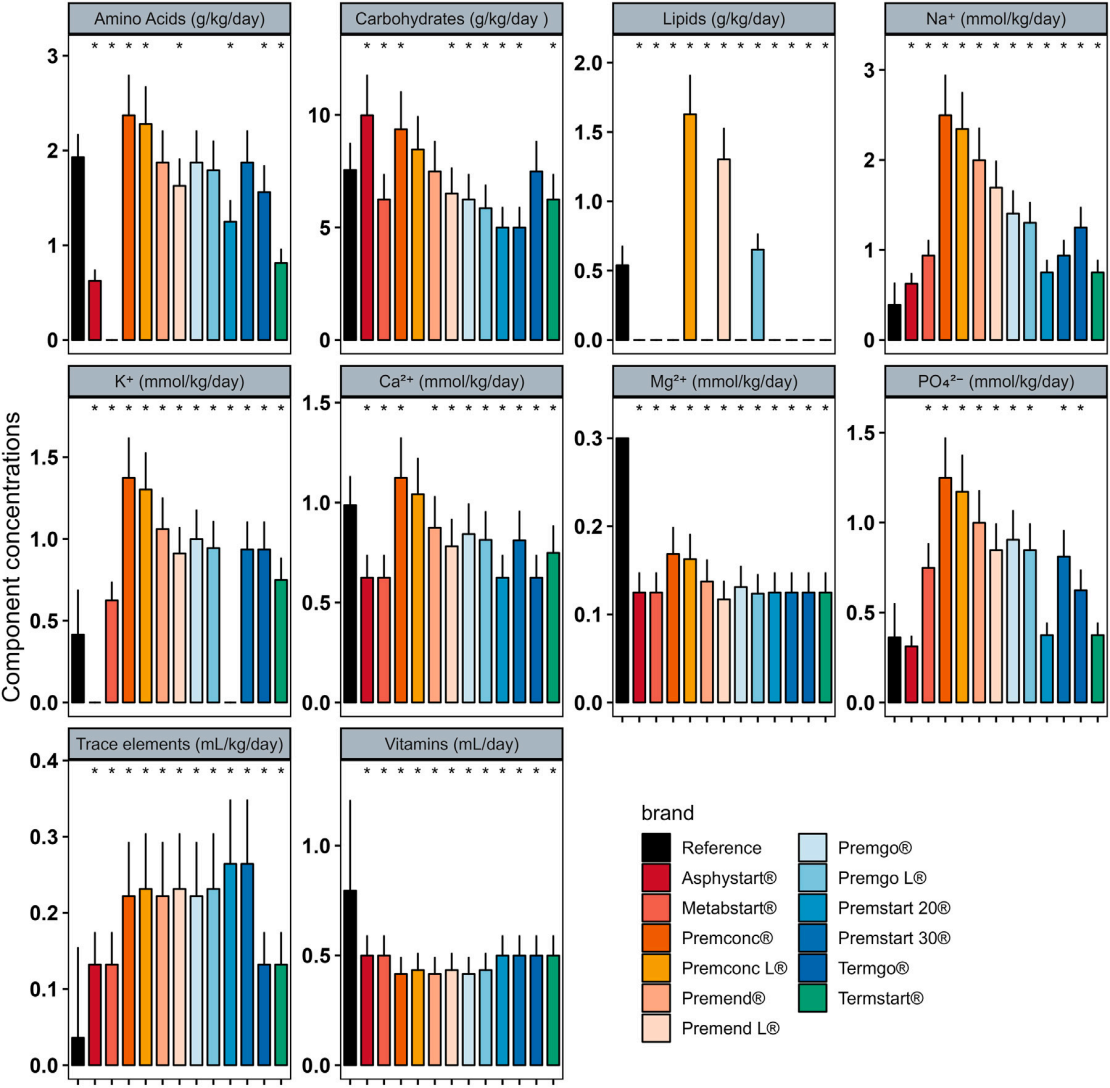
Mean (± standard deviation) expected intakes for each component with standard formulas, taking into consideration the volume ordered with individualised PN (reference) for patients in Group D_0_. * adjusted p–value <0.05 between expected intakes provided by standard formulas and amounts ordered.

**FIGURE 3:**
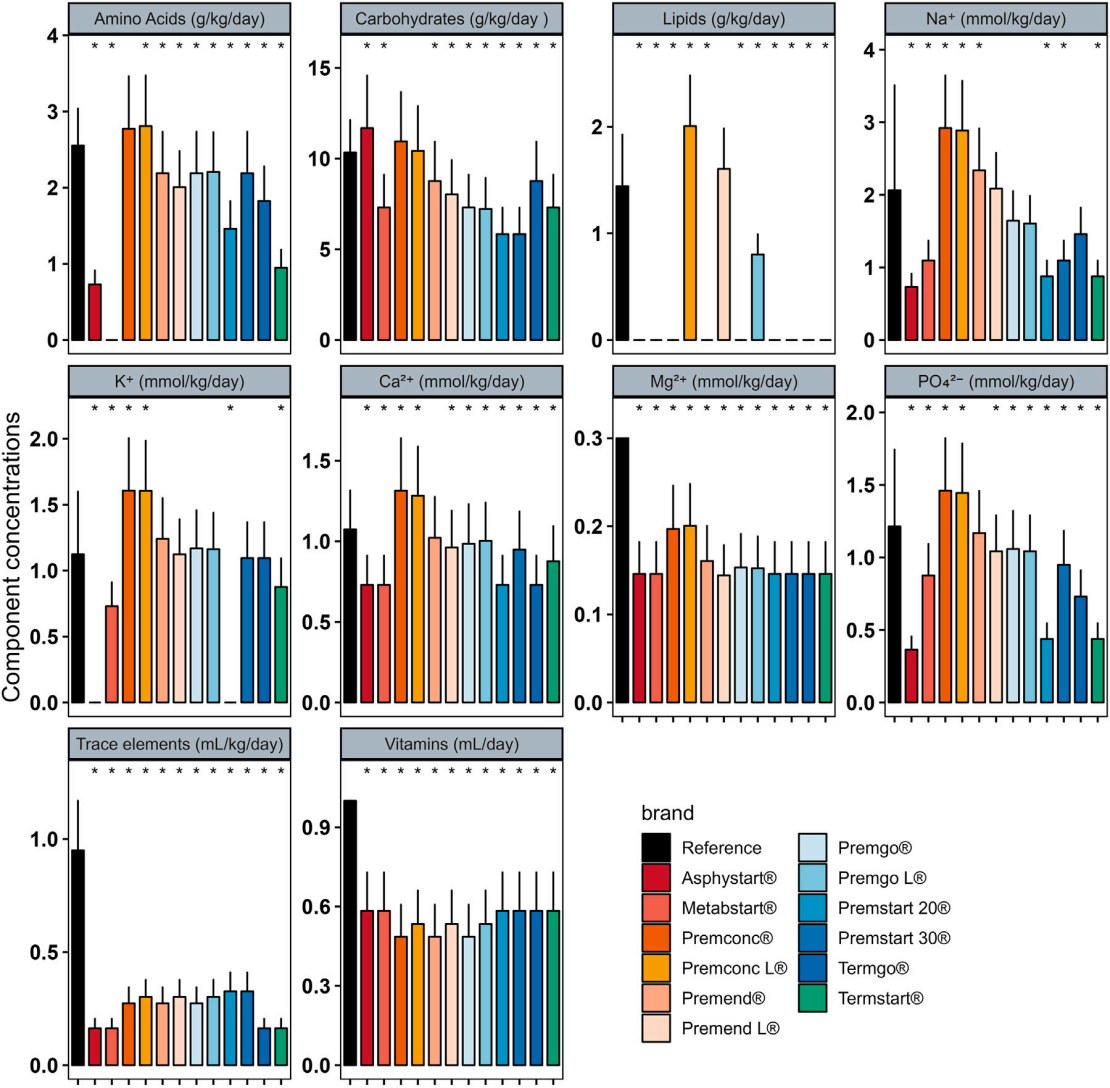
Mean (± standard deviation) expected intakes for each component with standard formulas, taking into consideration the volume ordered with individualised PN (reference) for patients in Group D_1–3._ * adjusted p–value <0.05 between expected intakes provided by standard formulas and amounts ordered.

**FIGURE 4:**
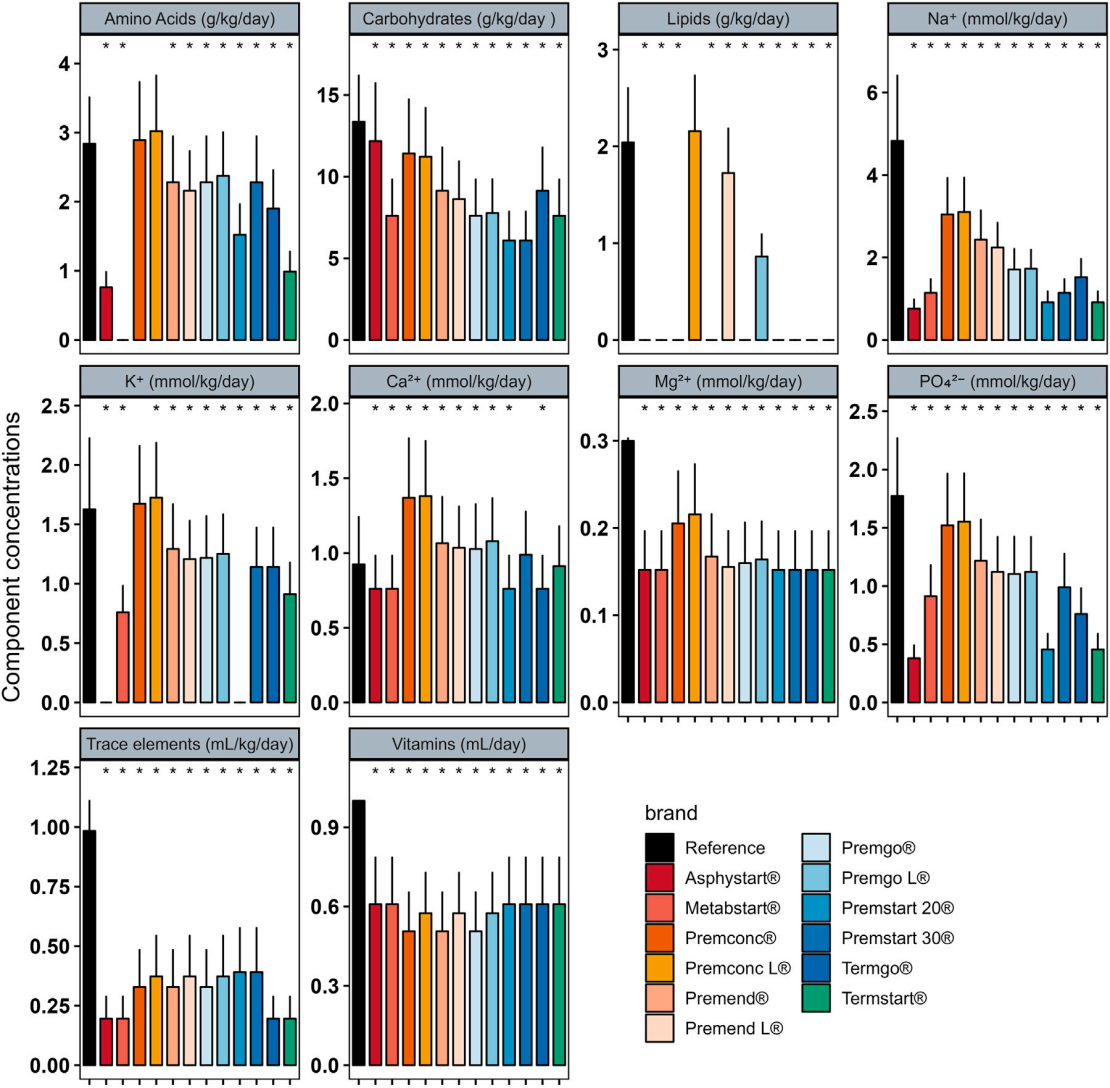
Mean (± standard deviation) expected intakes for each component with standard formulas, taking into consideration the volume ordered with individualised PN (reference) for patients in Group D_4+_. * Adjusted p–value <0.05 between expected intakes provided by standard formulas and amounts ordered.

In Group D_0_ there were significant differences between intakes ordered with individualised PN preparation and those that would have been provided by standard preparation for most components (Figure 2). Similar analyses were performed for Groups D_1–3_ (Figure 3) and D_4+_ (Figure 4).

**FIGURE 5:**
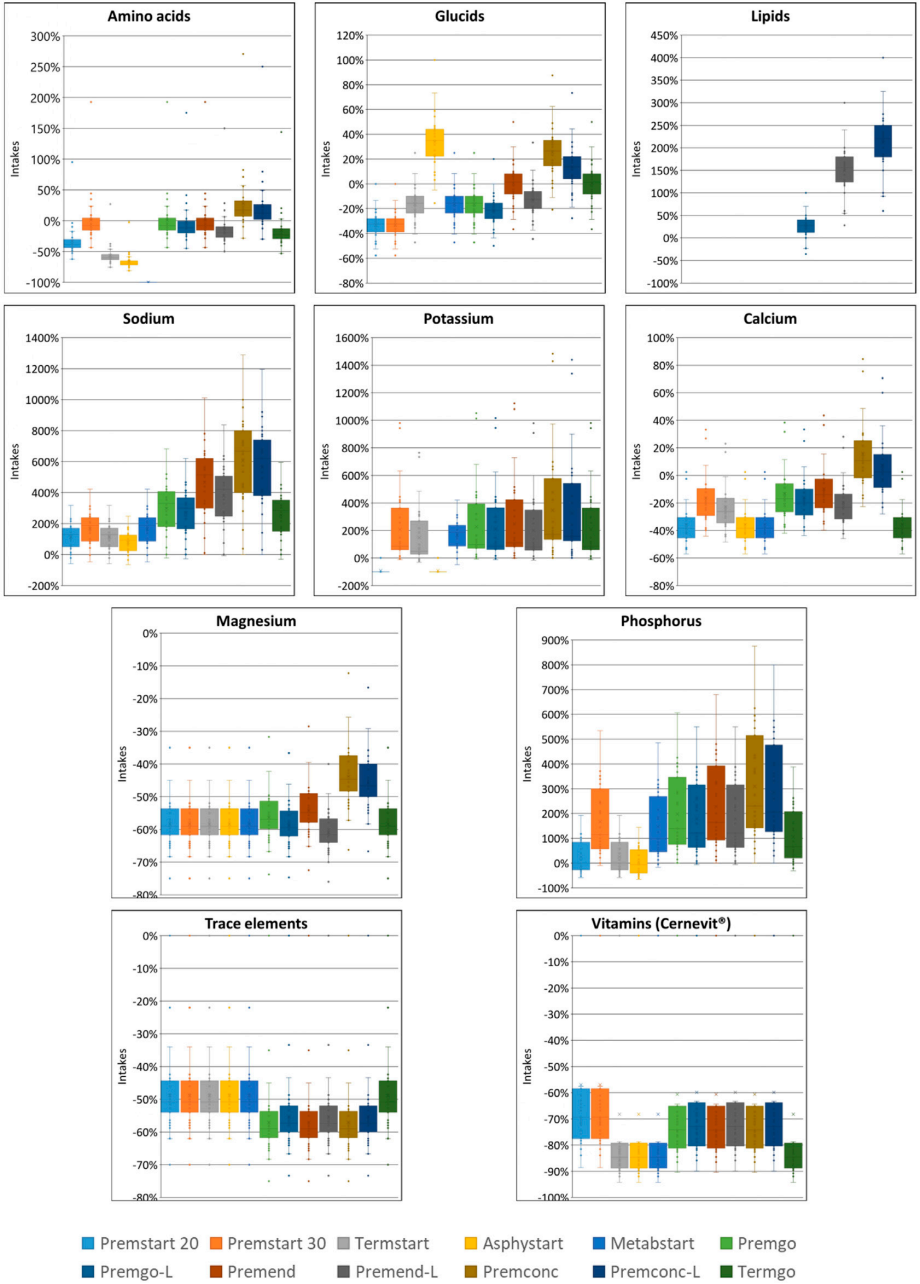
Box plots presenting the percentage of difference in intakes for each component of standard PN compared to those of individualised PN for the 12 standard formulas in Group D_0_. The volume of PN taken into consideration was the volume ordered in individualised PN. The box indicates the 25th to 75th percentiles, and the horizontal lines inside the box the median. Whiskers extend to the most extreme data points not considered outliers. Circles represent observations beyond the whisker length.

In group D_0_, sodium quantity in all standard formulas were consistently significantly higher than those delivered by individualized PN preparations, between 1.25 and 4 times more. The same was observed for potassium (almost double), with the exception of two standard formulations (Asphystart® and Premsart 20®), in which potassium was absent from the composition (Figure 2). Conversely, in the D_4+_ group, sodium, magnesium, phosphorus, trace elements, and vitamins provided by standard formulas was systematically lower than that achieved with individualized PN preparations, for example, the half for the magnesium (Figure 4). A similar pattern was observed in the D_1–3_ group for magnesium, trace elements, and vitamins (Figure 3). Although the amounts of amino acids and carbohydrates carried by standard preparation were mostly <20% of those ordered in individualised PN, no standard preparation had all components within this 20% difference in group D_0_ (Figure 5). Similar analyses were also performed for the two other Groups (D_1-3_ and D_4+_). Based on these two methods of comparison, no individualised preparation could be substituted by a standard one for all the three groups considered.

## Discussion

The present study found that none of the 12 standard formulas fit with targeted intakes achieved with individualised PN ordered for VLBW infants in our hospital. It is of note that all individualised PN prescriptions followed a strict written protocol, previously published [7], and which follows ESPGHAN, National Institute for Health and Clinical Excellence (NICE), and Australasian guidelines [5, 14–18]. The median volume prescribed in Group D_1-3_ was 79 mL/kg/day. This is in line with ESPHGAN guidelines for the first 3 days of life of premature infants [5] and with national guidelines in which the volume of preparation recommended in the initial and intermediate phases is between 80 and 120 mL/kg/day [12]. Regarding prescriptions for premature infants on D4 and beyond (Group D_4+_), the median volume prescribed for individualised preparations was 85 mL/kg/day (without taking enteral intakes into account), corresponding to the volume recommended by national and international guidelines [5, 12]. During the stabilisation phase in premature infants the recommended volume is comprised between 80 and 160 mL/kg/day and depends on whether there is parallel enteral feeding or not [5, 12]. For Group D_0_ (day of birth), the median volume prescribed was 64 mL/kg/day which is lower than recommended for the standard formulas. This may explain why the expected intakes with the standard formulas were systematically lower, and is in relation to the birthweight distribution as the majority of PN preparations were prescribed to ELBW infants. This highlights once again how it is difficult to establish national standard formulas for VLBW neonates, which is an heterogenous group. This is further compounded by the protocol for PN used in our hospital that requires the volume prescribed is reduced by 10–15 mL/kg/day below the expected daily fluid intake during the first 24 h as this volume is required on an arterial umbilical catheter.

Standardisation in PN could be helpful to initiate PN immediately after birth; in particular in terms of amino acids as it has been demonstrated that standard PN can enhance intake of amino acids compared to individualised PN in infants between days 1 and 5 of life, and is associated with improved gain of weight and head growth [19, 20]. As demonstrated by the analysis presented herein, in Group D_0_, only four bags designed by the national consortium provided for the amino acids needs (Premstart 30®, Premgo®, Premgo-L®, and Premend®). Although Premstrat 30® (originally designed for the initiation of parenteral nutrition in neonates) most closely resembles our individualised PN preparation, none of the components other than the amino acids present in these PN bags fitted with intakes provided by individualised PN. Furthermore, sodium and potassium intakes (with the exception of two formulas) were systematically greater than that provided by individualised PN preparation, making the use of a standard formula impossible because of the associated risk of hypernatremia/kalemia. In contrast, magnesium, trace element and vitamin intakes, irrespective of the group considered, were systematically lower with standard PN. As supplementation of elements to standard formulas is not allowed, the only option to use standard formulas in such cases would be a “Y-administration”. Nevertheless, this is a single-centre comparison between standard PN formulas proposed by a national consortium and individualised PN preparations. It is of note that the protocol used in our hospital for parenteral and enteral feeding [7] has been found to minimise post-natal growth restriction, even in high-risk patients such as ELBW infants [7, 8], extreme preterm infants [21], and those requiring post-natal steroid treatment or weaning from respiratory support [22]. In the protocol developed in our hospital, some elements are prescribed according a fixed daily dose. For example, magnesium is prescribed as 0.3 mmol/kg/day, whereas in standard PN formulas, concentrations vary according to the nutritional phase considered during the design of the bag. The authors of an Australasian study, based on an analysis comparable to that undertaken in our study, concluded that standardisation improved nutrient intake [23]. Although VLBW infants were also included in the latter study, it should be noted that the population characteristics differed: both gestational age and birth weight were higher than those observed in the cohort described herein. In addition, the Australasian standardised PN formulas were not identical to those used in France [12, 23]. For example, the formulation intended for the first day of life contained higher concentrations of carbohydrates compared with Premstrat 30®, and higher levels of both amino acids and carbohydrates than Premstrat 30®. Conversely, the Australasian formulations contained lower concentrations of electrolytes (particularly sodium and potassium), more closely aligning with our local protocol [7].

Many recommendations suggest using standard preparations and saving individualised for complex situations (such as metabolic disturbance, abnormal fluid or electrolyte losses, prolonged PN) [6], as they limit risks to the pharmaceutical supply chain [19, 24, 25]. In this way, standard PN preparations save time that can then be used for compounding individualised preparations when absolutely necessary. However, the present study highlights the importance of investigating whether or not standard PN formulas can be used. It is also of note that only 2.3% of these individualised PN preparations correspond to prescriptions for the day of the child’s birth. This is due to constraints related to the opening hours of the pharmaceutical production units, and this major drawback has been highlighted in other hospitals [26]. When the hospital pharmacy is unavailable to compound PN, they are prepared directly in NICU. In the latter, the quality of PN compounded is generally lower than in a pharmacy department, and non-conform preparation can be administered to patients [27, 28]. In this context, even if standard PN preparation do not fully reach neonate’s needs, it appears important to dispose of standard PN preparations, available in NICU at any time. According to the characteristics of neonates admitted to NICU, standard PN formulas should be national ones or locally adapted to fit the average neonate’s needs.

## Conclusion

In the present study, none of the 12 standard PN formulas proposed by the national consortium was adapted to substitute the individualised PN preparation compounded daily by the pharmacy. This can be attributed to the characteristics of the infants managed in the NICU. As standardisation in PN is helpful to initiate PN immediately after birth, the development of local standard formulas based on the department’s practices seems therefore relevant.

## Data Availability

The raw data supporting the conclusions of this article will be made available by the authors, without undue reservation.
